# Uterine Tumor Resembling Ovarian Sex-Cord Tumors Initially Diagnosed as a Prolapsed Fibroid

**DOI:** 10.1155/2018/4703521

**Published:** 2018-07-19

**Authors:** Fernando Augusto Rozário Garcia, Vanessa Pereira Gaigher, Rodrigo Neves Ferreira, Antônio Chambô Filho

**Affiliations:** ^1^Medical Resident, Department of Obstetrics and Gynecology, Hospital Santa Casa de Misericórdia de Vitória, Vitória, ES, Brazil; ^2^Pathologist, Hospital Santa Casa de Misericórdia de Vitória, Vitória, ES, Brazil; ^3^MD, PhD, Full Professor, Department of Obstetrics and Gynecology, Escola Superior de Ciências, Santa Casa de Miserícordia de Vitória, ES, Brazil; ^4^Head of the Department of Obstetrics and Gynecology, Hospital Santa Casa de Misericórdia de Vitória, Vitória, ES, Brazil

## Abstract

**Background:**

First described in 1945 by Morehead and Bowman, uterine tumors resembling ovarian sex-cord tumors (UTROSCT) are rare tumors of the uterine body that tend to occur in menopausal women presenting with abnormal vaginal bleeding, abdominal pain, and increased uterine volume. UTROSCT are usually diagnosed from incidental histological findings following hysterectomy performed due to a suspected endometrial polyp or uterine fibroids.

**Objective:**

To report on a 46-year-old patient with abnormal vaginal bleeding. At physical examination, a pediculated nodular lesion was found protruding from the external cervical os. Histopathology of the resected lesion led to a diagnosis of UTROSCT. Total abdominal hysterectomy with bilateral adnexectomy was then performed. The patient is currently undergoing regular outpatient follow-up, with no evidence of disease after one year.

**Methods:**

Data were retrieved from the patient's records, and macroscopic and microscopic images of the tumor were obtained.

**Discussion:**

Reports of metastasis or recurrence are rare. UTROSCT are considered of uncertain malignant potential and no particular form of treatment is formally recommended, with hysterectomy currently being the treatment of choice. This patient will be followed up for five years during which clinical examination and tomography of the chest, abdomen, and pelvis will be performed annually.

## 1. Introduction

Uterine tumors resembling ovarian sex-cord tumors (UTROSCT) represent a rare form of tumors that affect the uterine body [1]. Up to the present moment, fewer than 100 cases have been reported in the literature [2].

Morehead and Bowman published the first descriptions of UTROSCT in 1945 [3]. In 1976, Clemente and Scully studied these tumors and classified them into two groups: Group 1: endometrial stromal tumors with sex-cord-like elements (ESTSCLE) and Group 2: UTROSCT [4]. Diagnosis is reached following histopathology, with immunohistochemistry playing a crucial role, particularly with respect to the differential diagnosis [5].

Clinically, these tumor types generally occur in menopausal women, who may present with symptoms of abnormal vaginal bleeding, abdominal pain, and increased uterine volume [6]. Treatment options include hysterectomy with bilateral salpingo-oophorectomy or even hysteroscopic resection of the tumor; however, the management, prognosis, morbidity, and mortality associated with this pathology remain the subject of debate due to the scarcity of such cases in current literature [7].

This paper describes the case of a 46-year old patient with abnormal vaginal bleeding. She presented with a pelvic mass, identified at histopathology as a UTROSCT. Her follow-up and treatment after diagnosis are discussed.

## 2. Case Presentation

A 46-year-old, brown-skinned woman with regular menstrual cycles and one child presented at the gynecology department of a philanthropic hospital in Vitória, Espírito Santo, Brazil, reporting a 6-month history of intense vaginal bleeding associated with abdominal pain. She had suffered vaginal discomfort over the previous week. She had no prior history of allergy, comorbidities, use of medication, or surgery. There was no family history of gynecological cancer. At physical examination, she was found to be in good general health, alert, pale, with a flaccid abdomen, and no signs of peritoneal irritation. A hypogastric mass was detected. There were no vulvar lesions. Speculum examination showed no lesions in the vagina but revealed the presence of a bleeding mass extruding from the external cervical os and associated with intense bleeding at manipulation. At bimanual pelvic examination, it was possible to palpate the pedicle of the lesion through the cervical os.

In view of the initial diagnostic hypothesis of a prolapsed fibroid, vaginal myomectomy was performed. There were no complications following surgery and the patient was discharged from hospital the following day in good clinical conditions.

**Figure 1:**
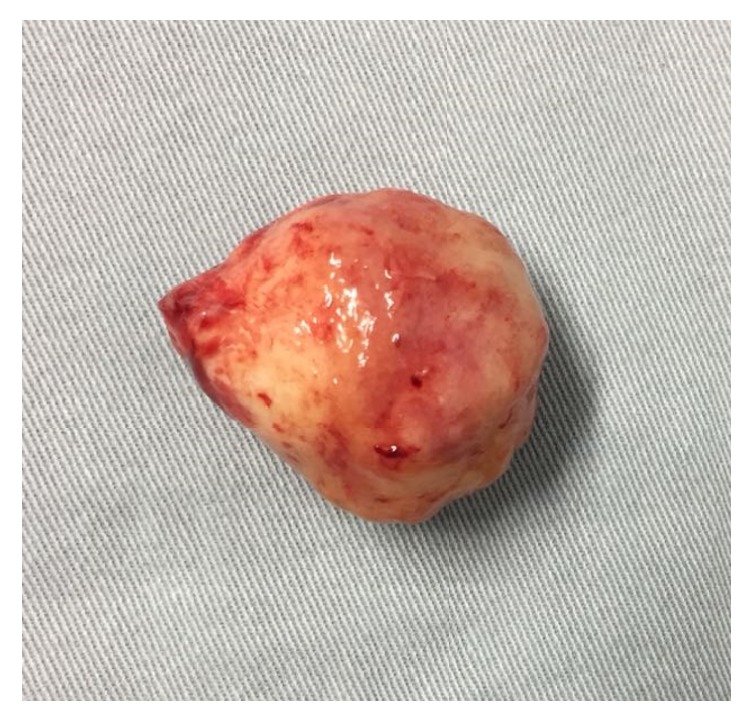
UTROSCT removed from the patient's uterus.

**Figure 2:**
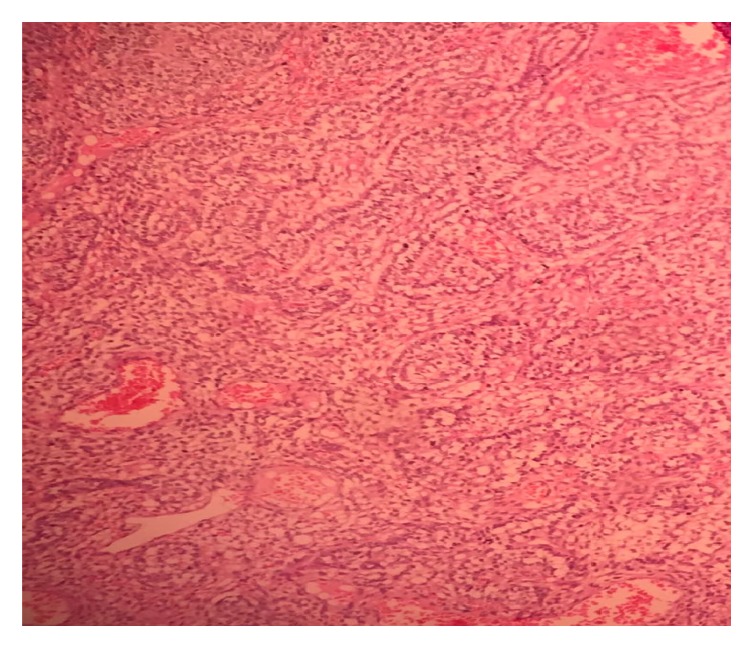
Hematoxylin-eosin (HE) staining, magnification 400x, clear cell cords resembling Sertoli cells.

**Figure 3:**
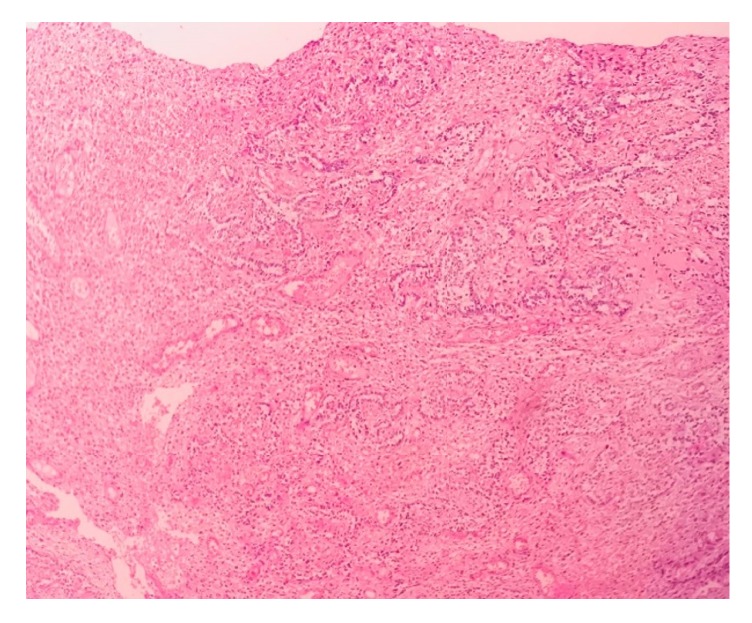
Hematoxylin-eosin, magnification 200x, clear cell cords resembling ovarian sex cord.

Macroscopically, the pinkish-colored nodule measured 3.5 x 3 x 4 cm (Figure 1). Microscopically, it consisted of a proliferative spindle cell nodule with gland-like, epithelioid, trabecular, and glomeruloid elements, without atypia. In some parts, the cells formed clear cell cords resembling ovarian sex cords. The core was rounded and normochromatic, and the cytoplasm was clear, resembling Sertoli cells. The stroma was partially hyalinized, resembling smooth muscle strips. There was no sign of necrosis and the mitotic index was low (2 mitoses/20 high-power fields) (Figures 2 and 3).

Immunohistochemistry confirmed the diagnostic hypothesis of a UTROSCT, with positive expression for CD56, smooth muscle actin, CD10, desmin, and pan-cytokeratin. The immunohistochemical markers for calretinin and inhibin were negative (Table 1).

The patient was readmitted to the department and metastatic screening was performed using computed tomography (CT) of the abdomen and chest. No abnormalities were found in the chest CT results. However, abdominal imaging showed the uterus to be greatly increased in size, with heterogeneous enhancement, an image resembling a cyst, probably of ovarian origin, in the left parauterine region, and a small amount of free fluid in the pelvis. No other abnormalities were found.

Serum levels of carcinoembryonic antigen (CEA) and CA 125 were <50 ng/ml and 15.50 U/ml, respectively.

Once the possibility of implantation metastasis had been ruled out, it was decided to submit the patient to a total abdominal hysterectomy with bilateral salpingooophorectomy. When accessing the abdominal cavity, a small amount of ascites was found and the left ovary was increased in volume, with a cystic appearance. The cecal appendix was normal. There were no complications following surgery and the patient was discharged from hospital the following day.

Histopathology performed on the surgical specimen obtained during the latest surgical procedure revealed uterine fibroids and focal adenomyosis, an endometrium with simple hyperplasia and no atypia, and a cervix with chronic cervicitis and squamous metaplasia. The right ovary was normal, while a hemorrhagic corpus luteum cyst was found on the left ovary. There was no sign of any residual UTROSCT.

The patient has been followed up regularly at the gynecological oncology outpatient department and remains asymptomatic one year after the second surgery. She will continue to be followed up as an outpatient for five years, with clinical examination and tomography of the chest, abdomen, and pelvis performed annually. At the end of the follow-up period, she will continue to undergo annual gynecological and clinical examination.

## 3. Discussion

A 46-year-old patient with abnormal vaginal bleeding underwent a surgical procedure to remove a pediculated nodular lesion protruding from the external cervical os. Histopathology led to a diagnosis of UTROSCT. Total abdominal hysterectomy with bilateral adnexectomy was then performed. Uterine fibroids and focal adenomyosis were identified in the surgical specimen, with no findings of residual UTROSCT. The patient is currently undergoing regular outpatient follow-up, with no evidence of disease after one year.

In 1976, Clemente and Scully classified these tumors into two groups: Group 1: ESTSCLE and Group II: UTROSCT. In ESTSCLE, less than 50% of the total tumor mass is composed of structural areas resembling sex cords and the tumor behavior is malignant in around 15% of cases [4, 5]. On the other hand, in UTROSCT, more than 50% of the total tumor mass resembles sex cords and in the great majority of cases its behavior is benign; however, there are cases in the literature involving metastasis and recurrence [7].

As in the case reported here, UTROSCT are more common between the fourth and sixth decades of life, with the principal symptoms being abnormal uterine bleeding and pelvic pain. Because of the rarity of this type of tumor, its incidence and mortality rates remain unknown [7].

Diagnosis can only be confirmed by histology after tumor resection, in most cases incidentally following hysterectomy performed because of a suspected endometrial polyp or uterine fibroids. Up to the present time, no noninvasive diagnostic tests such as specific serum markers or imaging findings have become available [6]. Although the potential for malignancy is low, there have been reports of UTROSCT associated with lymph node and extrauterine metastases [8].

Microscopically, these tumors are well demarcated; however, in rare cases they can be infiltrated between muscle fibers and may present with different microscopic forms such as sheets, tight nests, thin cords, trabeculae with wide interconnections, irregular islands, and hollow or solid tubules, with varying amounts of fibrous or hyalinized stroma. Cells with small amounts of clear or foam eosinophilic cytoplasm remaining from Sertoli and granulosa cells may be present. Typically, mitotic indexes are low with this type of tumor. It should also be emphasized that neoplastic endometrial stroma should not be found in the histological analysis of UTROSCT [9]. Differential histological diagnosis should be made between epithelioid and plexiform leiomyomas, uterine sarcoma, endometrial carcinoma, and metastatic sex-cord tumors of the ovary [10].

The tumor site varies and it may be submucous, intramural, or subserous. In the present report, the tumor was submucous, since pathological analysis of the uterine body showed no sign of the tumor after it had been removed [8]. Immunohistochemistry aids diagnosis, since the tumor shows positivity for the sex-cord markers calretinin, inhibin, CD99, Wilms' tumor protein 1, and melanoma antigen recognized by T cells (MART-1), for the epithelial markers pan-cytokeratin and epithelial membrane antigen (EMA), for the myeloid markers smooth muscle actin (SMA), desmin, and histone deacetylase 8, and for a range of other markers such as CD10, estrogen receptors, progesterone receptors, S100, and CD117, characterizing the varying phenotypes of this rare form of tumor [8].

There are few reports of metastasis and recurrence in the literature. In a systematic review published in 2018 that evaluated prognostic factors of recurrence in UTROSCT, recurrence rates of 6.3% were found for the 79 patients evaluated. Disease-free survival is high and is associated only with the type of surgery: 86% for patients submitted to resection of the tumor alone and 96% for those submitted to total hysterectomy, both for a five-year period [11]. The UTROSCT is considered of uncertain malignant potential, and there are no formal recommendations regarding treatment [7]. Up to the present time, hysterectomy has been the treatment of choice. Nevertheless, there are reports of some individual cases in which the patient wanted to go on to have children and the tumor was resected at hysteroscopy [7].

Although there is currently no consensus with respect to the follow-up regimen for cases of UTROSCT, the patient in the present report is undergoing annual outpatient follow-up, with no signs of metastasis or tumor recurrence twelve months after surgery. She will continue to be evaluated for five years, with clinical examination and tomography to assess for metastases in abdominal and pelvic organs.

This is the first reported case of a UTROSCT in a prolapsed fibroid protruding into the vagina through the cervix. The initial surgical procedure performed (vaginal myomectomy) would have been sufficient to completely resect the tumor in this case, since histopathology following the second procedure (total abdominal hysterectomy and bilateral adnexectomy) failed to detect any signs of residual UTROSCT, confirming the low malignant potential of this type of tumor.

## Figures and Tables

**Table 1:** Immunohistochemical analysis revealing a uterine tumor resembling ovarian sex-cord tumors (UTROSCT), Hospital Santa Casa de Misericórdia de Vitória, Vitória, ES, Brazil.

**Markers**	**Tumor expression**
CD56 – N-CAM	Positive
(clone 123C3)	
Smooth muscle actin	Positive
(clone 1A4)	
Pan-cytokeratin	Positive (multifocal)
(clone AE1/ AE3)	
Calretinin	Negative
(clone CRT01)	
Inhibin	Negative
(clone R1)	
CD10 (CALLA)	Positive
(clone SS2/ 36)	
Desmin	Positive (multifocal)
(clone DE-R-11)	
